# Assessment of cutting time on nutrient values, *in vitro* fermentation and methane production among three ryegrass cultivars

**DOI:** 10.5713/ajas.19.0369

**Published:** 2019-10-21

**Authors:** Chunmei Wang, Fujiang Hou, Metha Wanapat, Tianhai Yan, Eun Joong Kim, Nigel David Scollan

**Affiliations:** 1 State Key Laboratory of Grassland Agro-Ecosystems, Key Laboratory of Grassland Livestock Industry Innovation, Ministry of Agriculture and Rural Affairs, College of Pastoral Agriculture Science and Technology, Lanzhou University, Lanzhou 730020, Gansu, China; 2 Tropical Feed Resources Research and Development Center (TROFREC), Department of Animal Science, Faculty of Agriculture, Khon Kaen University, Khon Kaen 40002, Thailand; 3 Agri-Food and Biosciences Institute, Hillsborough, Co Down BT26 6DR, United Kingdom; 4 Department of Animal Science, Kyungpook National University, Sangju 37222, Korea; 5 Institute for Global Food Security, Queens University Belfast, Stranmillis Road, Belfast BT9 5AG, United Kingdom

**Keywords:** Diurnal Cutting Time, *In vitro* Dry Matter Digestibility, Methane, Nitrogen, Water Soluble Carbohydrates

## Abstract

**Objective:**

The 3×3 factorial arrangement was used to investigate if either high water-soluble carbohydrates (WSC) cultivars or suitable time of day that the grass cut could improve nutrient values and *in vitro* fermentation characteristics.

**Methods:**

The 3 cultivars were mowed at 3 diurnal time points and included a benchmark WSC ryegrass cultivar ‘Premium’, and 2 high WSC cultivars AberAvon and AberMagic, which contained, on average, 157, 173, and 193 g/kg dry matter (DM) of WSC, and 36.0, 36.5, and 34.1 g/kg DM of N during 7th regrowth stage, respectively. The fermentation jars were run at 39°C with gas production recorded and sampled at 2, 5, 8, 11, 14, 17, 22, 28, 36, and 48 h. The rumen liquid was collected from 3 rumen fistulated cows grazing on ryegrass pasture.

**Results:**

High WSC cultivars had significantly greater WSC content, *in vitro* DM digestibility (IVDMD) and total gas production (TGP), and lower lag time than Premium cultivar. Methane production for AberMagic cultivar containing lower N concentration was marginally lower than that for AberAvon and Premium cultivars. Grass cut at Noon or PM contained greater WSC concentration, IVDMD and TGP, and lower N and neutral detergent fiber (NDF) contents, but CH_4_ production was also increased, compared to grass cut in AM. Meanwhile, the effects of diurnal cutting time were influenced by cultivars, such as *in vitro* CH_4_ production for AberMagic was not affected by cutting time. The IVDMD and gas production per unit of DM incubated were positively related to WSC concentration, WSC/N and WSC/NDF, respectively, and negatively related to N and NDF concentrations.

**Conclusion:**

These results imply either grass cut in Noon or PM or high WSC cultivars could improve nutrient values, IVDMD and *in vitro* TGP, and that AberMagic cultivar has a slightly lower CH_4_ production compared to AberAvon and Premium. Further study is necessary to determine whether the increase of CH_4_ production response incurred by shifting from AM cutting to Noon and/or PM cutting could be compensated for by high daily gain from increased WSC concentration and DM digestibility.

## INTRODUCTION

As one of the most important and common forages for dairy cows in temperate regions [1], ryegrass widely sown for grazing ruminants (*Lolium perenne* L.) is featured by high forage yield and nutritive value, especially high soluble and degradable N and carbohydrates [2]. Ruminant production is moving towards rely on more forage or only grass-based diets, but the grass-based system is challenged by the large fluctuation in grass availability and nutrient values affected by seasonal condition and growth period. The quality of grass has effect on nutrient intake and utilization efficiency [3,4], and the problem of poor-quality forages leaded to low microbial protein yield cannot simply be solved or completely compensated by supplementing high amounts of concentrates [5]. It is also revealed that good quality grass has the potential to sustain high feeding efficiency similar to concentrates [6], which could reduce farming cost. Hence, identifying the nutritive values of perennial ryegrass is critical for ruminant production system.

Previous studies indicated that increasing water-soluble carbohydrates (WSC) concentration in diets could improve daily live weight gain [7], dry matter (DM) digestibility and milk yield [3,8], reduce CH_4_ emission per unit of DM intake and live-weight gain [7] by favoring the rumen fermentation with propionate and butyrate production [8,9], and decrease nitrogen excretions especially urinary N excretion in ruminant farming [10,11] by increasing the capture of ammonia in rumen and utilization efficiency of endogenous N cycle [10,12] and promoting microbial protein synthesis [9,13]. Nutritive values (e.g., WSC) of grass are not only influenced by inherited characteristics such as improved cultivars and genetic selection, but also determined by short physiological responses of the plants to changes in their immediate environment such as diurnal cutting management [3,14]. In rotational grazing systems, cows transformed into a new perennial ryegrass paddock in the evening [15] and/or offered alfalfa cut in PM [3] had greater milk yield. The response of nutrient values to diurnal cutting variations and grass cultivars is necessary to be identified for optimizing grazing management. Furthermore, interaction effects of diurnal cutting time and cultivar on nutritive values and *in vitro* gas production are still limited data available. Hence, this study was to investigate the effects of cultivar and diurnal cutting time on chemical compositions, *in vitro* dry matter digestibility (IVDMD) and in *vitro* gas production parameters.

## MATERIALS AND METHODS

All procedures involving animals were conducted under the regulations of UK Home Office Animals (Scientific Procedures) Act, 1986.

### Experimental design

The present study was designed as a 3 (perennial ryegrass cultivars) × 3 (diurnal cutting times) factorial design experiment at Plas Gogerddan, Aberystwyth, UK (52°25′52.1″N, 4°01′01.8″W). The 3 grass cultivars included a benchmark WSC perennial ryegrass cultivar ‘Premium’ and 2 high WSC cultivars AberAvon and AberMagic. The 9 plots for 3 cultivars (3 plots for each cultivar) were arranged in a randomized block design, and each plot was 12.5 m×1.2 m (15 m^2^). Before the beginning of the experiment, ryegrass sward was trimmed throughout at a residual height of 4 cm with a Haldrup harvester, and then allowed to re-grow 3 wk to reach the height of ca. 20 cm in simulation to grazing condition. Five quadrats (0.5 m×0.5 m) were taken in a “W” shape across each plot in 7th regrowth for a consecutive 3 d from 11 to 13 September. Diurnal cutting time was 0700 (AM, half hour after sun rise; GMT+1 in UK), 1330 (Noon, the strongest radiation), and 1900 (PM, half hour before sunset) in September, respectively. The grass samples taken from 5 quadrats in each plot in a consecutive 3 d at the same cutting time were pooled into one sample for each plot. Samples freeze-dried at −20°C were ground through 1 mm sieve screen, and then divided into 2 subsamples. One portion was used for the measurement of *in vitro* gas production, and another was used for determining chemical compositions.

### *In vitro* fermentation measurement

Fermentation systems were consisted of 30 (27 samples + 3 blanks without forage substrate) 160 mL air tight serum bottles (Phase Separations Ltd., Clwyd, UK). All bottles were incubated in a water bath at a constant temperature of 39°C and maintained in an anaerobic environment. Incubations were repeated when fermentation parameters for the same treatment deviated by more than 10% from average value.

Rumen fluid was taken from 3 fistulated cows grazed in perennial ryegrass pasture in morning. Mixed rumen fluid preserved in a pre-heated vacuum (39.5°C) flask was rapidly transported into laboratory (within 1 h) and then strained through a double layer of muslin. All laboratory handling of rumen fluid was carried out under a continuous flow of CO_2_.

Each bottle contained 95 mL mixture of buffer solution and rumen fluid (2:1, v/v) and 1.0 g ground dry forage substrates, except for 3 blank bottles with no forage substrate added. The buffer solution was formulated by Menke and Steingass [16]. A mixture of rumen fluid, buffer solution and ground forage substrates was maintained under an anaerobic environment. Pressure and volume for gas production in each bottle were recorded at 2, 5, 8, 11, 14, 17, 22, 28, 36, and 48 h, as well as sampling gas, respectively. Gas sample was collected using a plastic gas collection bag attached to serum bottle through a 3-way syringe valve, and finished while reading of pressure transducer is zero (ambient pressure). Bottles were stored at 4°C in the end of fermentation. For determination of DM loss, the residues in the bottle were vacuum-filtered through a weighed glass microfiber filter (1.6 μm) on a porcelain funnel and rinsed twice with distilled water (100 mL), and then placed into a grip-seal bag for DM content measured.

### Chemical analysis

The DM content was determined by freeze-dried at −20°C. Ash content was analyzed by combusting the ground samples at 550°C for 6 h on a muffle. Nitrogen content was analyzed by micro-Kjeldahl techniques using ‘Kjeltec’ apparatus (Perstorp Analytical Ltd., Maidenhead, Berkshire, UK). Neutral detergent fiber (NDF) concentration without alpha-amylase and sodium sulfite, expressed as inclusive of residual ash, was determined according to the method of Van Soest and Wine [17] using the Tecator Fibretec System equipment (Tecator Ltd., Thornbury, Somerset, UK). The WSC content was determined as described by Thomas [18]. Methane and carbon dioxide concentrations sampled from incubation vessels were analyzed by Infrared gas analysis (5000 series gas analyzer; Analytical Development Co. Ltd., Hoddesdon, Hertfordshire, UK).

### Statistical analysis

The analysis was carried out in SAS (version 9.2, SAS Institute Inc., Cary, NC, USA) with a probability level of 0.05 for significance of treatments and interaction. Generalize linear model procedure was used to evaluate the effects of cultivar and cutting time on nutrient values and gas production parameters with cutting time and cultivar as fixed factor, and Pearson Correlation procedure was implemented to describe the relationships between IVDMD and *in vitro* gas production parameters and linear and quadratic chemical compositions. Figures were made by Origin 2017 using the linear and binomial fitting and/or exponential fitting (OriginLab Corporation, Northampton, MA, USA).

The Gompertz function (Eq.1, [19]) was used to analyze total gas and CH_4_ production using the non-linier regression analysis program (NLREG), where GP_t_ is cumulative gas production (mL/g DM), A is the theoretical maximum of gas production (mL), b is the rate of gas production (mL/h), lag is lag time (h), t is the incubation time (h) and e is the constant. Mean prediction error (MPE, Eq.2) was used to describe the prediction precision [2], where M and P are the actual measured and predicted values, respectively, and n is the number of pair of values of P compared with M.

Eq.1 $${\text{GP}}_{\text{t}}=\text{A}\times \text{exp }\left\{-\text{exp }\left[1+\frac{\text{b}\times \text{e}}{\text{A}}(\text{lag}-\text{t})\right]\right\}$$

Eq.2 $$\text{MPE}=\sqrt{\frac{\Sigma {\left(\text{P}-\text{M}\right)}^{2}}{\text{n}}}/(\sum \text{M}/\text{n})$$

## RESULTS

### Effect of cutting time and cultivar on chemical compositions and *in vitro* dry matter digestibility

Neither cutting time nor cultivar affected organic matter (OM) content. The NDF content for grass cut in PM was lower (p<0.001) than that cut in AM, but the effect of cutting time depended on the cultivars such as the NDF content for AberAvon cut in PM was no significant difference with that for grass cut in AM (p>0.05). Both cutting time and cultivar had significant effects on N and WSC concentrations (p< 0.001). The WSC concentrations were increased when the cutting time was moved from AM to Noon and then to PM (p<0.001), but N concentrations were decreased (p<0.001). As expected, high WSC cultivars contained higher WSC concentration (p<0.001). Nitrogen concentration for AberMagic cultivar was significantly lower than that for AberAvon and benchmark cultivar (Table 1).

There were only main effects of cultivar and cutting time on IVDMD. As expected, both grass cut in Noon or PM (p< 0.001) and high WSC cultivars (p = 0.043) had greater IVDMD than that for grass cut in AM and/or benchmark cultivar. There was no significantly different in IVDMD for grass cut in Noon and PM (Table 1).

### Gas production and parameters of Kinetics function

**Figure 1:**
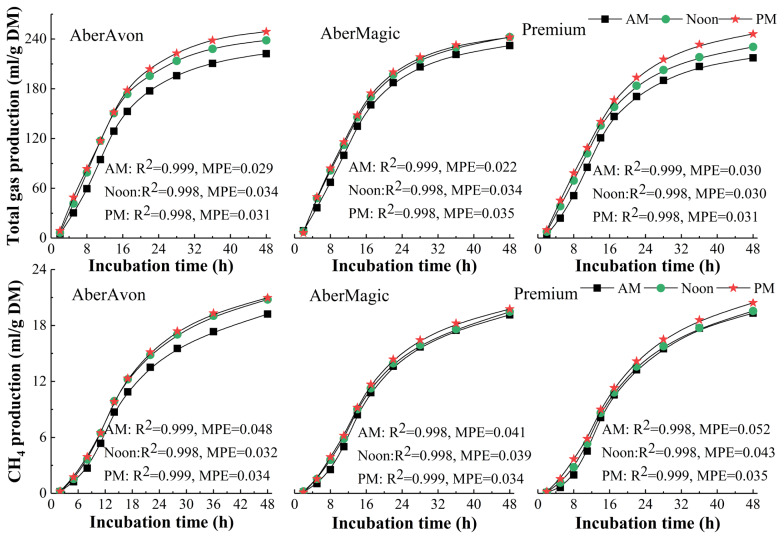
Cumulative total gas and CH_4_ production at various incubation times of 3 cultivars. MPE, mean prediction error; R^2^, the pseudo correlation coefficient.

*Kinetics function for gas production*: Gas production for the 3 cultivars rapidly increased from 2 to 17 h, the increment was faded down from 22 to 36 h and then the curves tended to be stable (Figure 1). The interaction of cutting time and cultivar affected cumulative total gas production (TGP, mL/g DM) only at 2 (p<0.01) and 48 h (p<0.05) for incubation, and affected CH_4_ production (mL/g DM) only at 2 h (p<0.001). Cutting time and cultivar both had significant effects on TGP from 2 to 48 h, except cultivar no affecting TGP at 2 h. The TGP and CH_4_ production for grass cut in Noon and PM were higher than that cut in AM. High WSC cultivars had greater TGP, except TGP for AberAvon was no difference with that for Premium at 2, 36, and 48 h. The CH_4_ production for Premium was numerically higher than that of AberMagic at all incubation time, and lower than that of AberAvon at 2 to 22, and 48 h. Methane production for grass cut in PM was greater than that cut in AM (p<0.05).

*Effects of cutting time and cultivar on in vitro gas production parameters*: Either cultivar or cutting time had significant effects on TGP parameters, except TGP/*in vitro* digestible DM incubated. Grass cut in Noon and PM had greater TGP, expressed as a proportion of DM, OM, and *in vitro* digestible DM incubated, A and b, and lag time was decreased (Table 2). High WSC cultivars had higher TGP parameters and b, and lower lag time, and all parameters for AberMagic cut in Noon and PM were no significantly different.

There were no interaction effects on CH_4_ production parameters, except lag time, and b was not affected by both factors (Table 2). Methane yield per unit of DM and OM incubated, and A for high WSC cultivars were slightly higher than that for benchmark cultivar, and high WSC cultivars had lower lag time. Grass cut in Noon and PM had higher CH_4_/DM incubated, CH_4_/OM incubated, and A. However, the effect of cutting time was determined by cultivar, such as AberAvon cut in Noon and PM contained higher CH_4_ production than that cut in AM, but that for AberMagic was not affected by cutting time.

**Figure 2:**
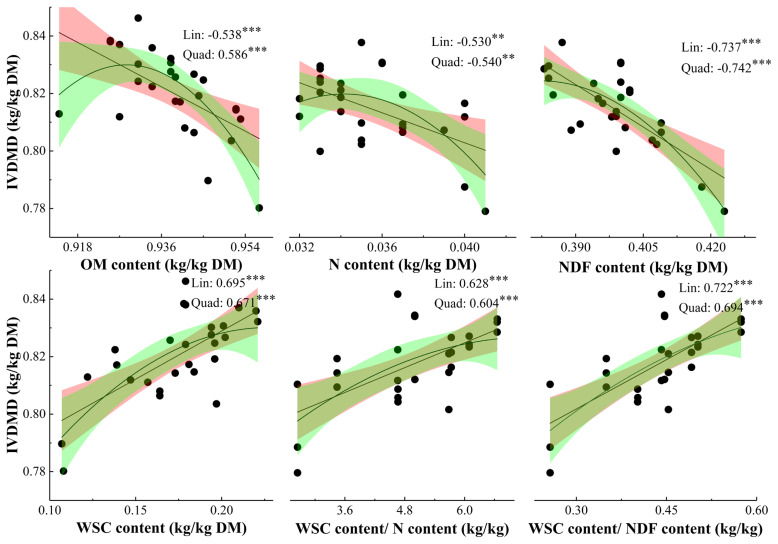
Relationship between chemical compositions and IVDMD for ryegrass. Lin, linear; Quad, quadratic. * p<0.05, ** p<0.01, *** p<0.001. Values represents Pearson correlation coefficient. IVDMD, *in vitro* dry matter digestibility; OM, organic matter; N, nitrogen; NDF, neutral detergent fiber; WSC, water soluble carbohydrates. The fitting lines represent linear and quadratic fits, and the shadows represent the range of 95% confidence interval.

*Relationships between chemical compositions and parameters for gas production*: The correlation coefficients derived from linear parameters were not lower than that from quadratic parameters, except N and NDF concentrations (Figure 2). The IVDMD was negatively related to OM, N, and NDF concentrations, and positively related to WSC, WSC/N, and WSC/NDF concentrations.

The relationships between nutrient composition parameters and *in vitro* fermentation parameters had higher correlation coefficients when the linear regression was fitted as linear, rather than as quadratic (not presented), except N, NDF, and NDF/OM concentrations. Neither CH_4_/*in vitro* digestible DM incubated nor b for CH_4_ production was correlated with any chemical composition parameter (Table 3). The TGP/DM incubated, CH_4_/DM incubated, A, and b of TGP, and A of CH_4_ production were all positively related to WSC, WSC/N, and WSC/NDF contents, and negatively related to N content. Meanwhile, lag time for TGP and CH_4_ was negatively related to WSC, WSC/N, WSC/NDF, and IVDMD, and positively related to N, NDF, and NDF/OM contents. The IVDMD had positive effect on TGP parameters, but had no effect on CH_4_ yield.

## DISCUSSION

### Effects on cutting time and cultivar on chemical compositions

The relative changes in WSC concentration also can be achieved either through improved cultivars and genetic selection or cutting management. With PM cutting, the WSC concentration of ryegrass was increased by up to 61 g/kg DM in present study, while genetic selection resulted in an increase up to 35 g/kg DM comparing with the benchmark cultivar. The WSC concentration in the present study harvested in middle of September were, on average, 173, 193, and 157 g/kg DM for AberAvon, AberMagic, and Premium, respectively, which were far lower than that (307, 302, and 269 g/kg DM, separately) for the same 3 cultivars harvested in late May and early June conducted in the same region [20]. The gap could be leaded by that grass utilized in September contains lower proportion of sheaths and higher proportion of stems, compared with that utilized in May [10], and also could be attributed to lower solar radiation and the air temperature in September [21]. When photosynthesis production in herbage exceeds carbohydrates utilization, WSC would be accumulated during the day [15,22], which was in line with that soluble carbohydrate content in Festuca was accumulated up until 1800 [23] and WSC concentration for alfalfa cut in PM was higher than that cut in AM [3]. The WSC contents for 3 cultivars were considerably increased from AM to Noon, while the rate of increment was slowly from Noon to PM, which might be due to lower net photosynthetic rate of ryegrass in PM than that in AM [22].

Other chemical composition concentrations probably varied in a passive manner among different cutting time by concentrations (e.g. OM) or dilutions (e.g. crude protein [CP] and NDF) effects within the accumulation of nonstructural carbohydrates [15,23,24]. There was a decrease of NDF concentration for AberMagic and Premium cut in Noon and PM, which was consistent with the studies of Lechtenberg et al [23], Ellis et al [12], and Smit and Elgersma [14]. In the absence of photosynthesis, total soluble carbohydrate content falls due to respiration during the night, which leads to an increase in protein synthesis [15]. The previous studies also reported consistent results, for example, ca. 50% of daily increment of sucrose was lost from sundown to the early morning of the 2400 and 63% of leaf starch increased between 6 am and 6 pm was lost between the early morning of the 2400 and 0300 [23]. The peak of CP concentration for alfalfa showed between 0300 and 0600, which gradually declined after sunrise [24]. The present study obtained a negative relationship between N and WSC content (Pearson correlation coefficient was −0.819, p<0.001), and grass cut in Noon and PM and high WSC cultivars contained higher WSC/N content. Hence, in present study, grass cut in Noon and PM cultivars contained greater WSC and lower N concentrations.

### *In vitro* dry matter digestibility

In current study, IVDMD for grass cut in Noon and PM was higher than that cut in AM, which was in line with the results of Burner and Belesky [25], and Alende [26]. The IVDMD for Noon and PM samples of ryegrass were 2.78% and 2.28% higher than AM samples, which was higher than an increase of 1.6% for alfalfa samples cut from 6 am to 6 pm [23]. Meanwhile, IVDMD for high WSC cultivars was greater than that for Premium, which was due to that microbial protein synthesis would be compensated by a decrease in dietary N supply, especially when rapidly and sufficiently available energy was used as the energy source [27]. As it was evidenced by that the decrease in ratio of N to soluble sugar improved the N utilization and milk yield [8], and the slightly higher ruminal pH for cows offered alfalfa cut in PM due to lower acetate concentration leaded to numerical greater DM digestibility than offered alfalfa cut in AM [3]. As showed in Figure 2, WSC/N and WSC/NDF all had positive relationships with IVDMD, which was agreed that NDF content had negative relationship with digestibility [2,28], and WSC content was positively correlated to DM digestibility [2]. Hence, both grass utilized in Noon and PM and high WSC cultivars have the potential to improve IVDMD.

In the present study, N content negatively related to IVDMD was not in line with the study of Stergiadis et al [2], which might be attributed to relatively higher N concentration ranging from 31.5 to 40.9 g/kg DM (197 to 256 g/kg DM for CP concentration) in present study. When the N concentration was 34.0 g/kg DM, the value of IVDMD predicted by N concentration reached the peak value in present study. The threshold value was close to 35 g/kg DM reported by Stergiadis et al [2], which was exhibited that increasing grass N content improved N digestibility when grass N content was below about 35 g/kg DM and any increase in grass N content over 35 kg/kg would decrease N digestibility. In present study, the response of IVDMD to N content shifted from no correlative to negative, after N content increased from below to beyond 34.0 g/kg DM, which could be explained by that nitrogen exceeding the N requirement for rumen microbial activities would produce excessive ammonia *in vitro*, and result in high N excretion especially in urine *in vivo* [11]. Hence, diet based on high WSC cultivars and grass cut in Noon and PM containing rich rapid available soluble sugar, and high solubility and degradability of fresh-grass N [10], where N content mainly was replaced by WSC content, could offer optimum N and energy for ruminant production, and have the potential to improve DM digestibility and reduce N excretion.

### Parameters of gas production

The present results showed that TGP was affected both cultivar and cutting time, and CH_4_ production was only affected by cutting time, which were in line with the results of Tang et al [29]. In the present study, all growth rates of gas production were firstly increased and then declined, which might be caused by the decrease of fermented OM and protein availability, and accumulation of volatile fatty acids (VFAs) during the process of fermentation. The TGP rapidly increased from 2 to 17 h, and CH_4_ production increased from 5 to 22 h of 48 h incubation, which were consistent with those reported by Lovett et al [30], but Tang et al [29] showed that relatively rapid fermentation rate was presented after 8 h for 48 h incubation. In present study, 52% to 65% of gas production occurred at the first 14 h and 54% to 59% of CH_4_ occurred at first 17 h, which had obviously lower fermentation rates than that from the study of Muck et al [31] that 56% to 70% of gas production occurred at 9 h for the 96 h incubation. A range of factors affect the fermentation rate, e.g., species of donor animals, diets fed to donor animals and nutritive values of fermentation substrates [32].

In the present study, high sugar cultivars contained greater WSC content, thus had faster fermentation rate (Tables 2 and 3), e.g., higher fermentation rates during the first couple of hours and lower lag time of gas production. However, the absence of WSC content affecting CH_4_ yield might be contributed by unexpected lower difference in WSC concentration of 3 cultivars, which was in line with the studies of Niderkorn et al [20] and Smit and Elgersma [11]. Meanwhile, Ellis et al [12] reported sugars might have a higher methanogenic potential than starch or even fiber, and lead to higher CH_4_ yield *in vivo* (kg/kg DM intake). By contrast, Lovett et al [30] and Kim et al [7] found that compared to low WSC cultivars, diet based on high WSC cultivars reduced CH_4_ yield, as a proportion of feed intake. In present study, either grass cut in noon and PM or high WSC cultivars was not effective way to lower CH_4_ yield. However, emission intensity in terms of products (e.g. weight gain and milk yield) would be potential to be lower [7], due to higher total and digestible DM yield per ha would be expected to result in growing faster and shorten cycle of slaughter.

The present study found that IVDMD, WSC, WSC/N, and WSC/NDF concentrations had positive relationships with TGP expressed as per g DM incubated, and A and b for TGP, but those were negative with the lag time for TGP, which was in line with the study of Tang et al [29]. The result implied that the high soluble carbohydrates was beneficial for rumen fermentation, and high N content without sufficient utilized energy leading to excess the requirement of microbial synthesis resulted in lower TGP due to excessive ammonia which has negative effect on TGP. The positive relationship between CH_4_ production and WSC content was in line with Ellis et al [12]. The NDF content was the most effective diet factor to reduce the rate of rumen fermentation, and NDF/OM was positively related to the ratio of acetate/VFAs and negatively related to the ratio of propionate/VFAs [28]. In the present study, grass NDF content had a negative relationship with TGP, but CH_4_ yield not affected by NDF content might be attributed to not large enough difference among NDF concentrations of all samples. The results imply that both increasing WSC content and improving DM digestibility are beneficial for rumen fermentation and the rumen microbial population [9,30]. Meanwhile, the synchronous supply of N and fermentable energy to the rumen is essential to maximize the microbial growth and consequently N utilization efficiency [33]. Hence, further studies are needed to quantity the interaction effect of N and WSC concentrations on gas production, especially the effect of WSC content on CH_4_ yield *in vivo*.

The relationships between gas production parameters and chemical compositions were different under different N level (Table 4). After N content changing from below to beyond 34.0 g/kg DM, the effect of N content on TGP yield was changed from no correlative to negative, and the respond of TGP yield to WSC content was changed from negative to positive. The results probably could be explained by that the high available fermented energy exceeding the requirement of microbial synthesis would contribute to lower pH, which is disadvantage to microorganism activity, when N content below 34.0 g/kg DM. Otherwise, the insufficient WSC could not offer enough fermentable energy for meeting the requirement for microbial activity, and excessive fermentable N would produce excessive ammonia while N content beyond 34.0 g/kg DM. In an *in vivo* experiment, it was reported that after N content reaching to 33.6 g/kg DM [34], an increase of N concentration had no effect on milk production and increase urinary N excretion for cows, and even the dietary N content could be decreased to ca. 24 g/kg DM without compromising milk protein N yields [35]. The difference probably attributes to the no passage out rate for this *in vitro* experiment, which leads to the accumulation of fermentation. The further studies are required for identifying the optimal proportion of WSC and N concentrations for maximizing entire tract nutrient digestibility and CH_4_ emission for ruminant production.

## CONCLUSION

Shifting AM cutting to Noon and PM cutting reduced N and NDF concentrations for ryegrass, and increase WSC content, IVDMD, TGP and CH_4_ yield, except CH_4_ yield for AberMagic cultivar was not significantly influenced. Compared to Premium cultivar, high WSC cultivars contained higher WSC content, IVDMD and TGP, and lower N concentration. Furthermore, AberMagic cultivar had slightly lower CH_4_ yield. These results reveal information and recommendations for farmers who engage in ruminants raising under zero-grazing to try to improve the cultivation of better varieties such as the high sugar varieties and/or to harvest grass at suitable time like during the mid-day, noon and/or afternoon to optimize grazing management, which would consequently increase DM digestibility by providing the optimum available WSC and N. The AberMagic cultivar also have the potential to reduce CH_4_ emission, but further study for the effect on animal production in conjunction with methane emission is necessary.

## Figures and Tables

**Table 1:** Effects of cutting time and cultivar on chemical compositions and IVDMD (n = 27)

Items	AberAvon			AberMagic			Premium			SEM	p-value		
			
	AM	Noon	PM	AM	Noon	PM	AM	Noon	PM		Cultivar	Cutting time	Interaction
OM (g/kg DM)	934	934	939	946	939	933	939	940	940	9.2	0.709	0.932	0.622
N (g/kg DM)	39.8^e^	35.5^cd^	34.1^bc^	35.9^d^	33.4^ab^	33.1^ab^	39.6^e^	36.2^d^	32.0^a^	0.79	<0.001	<0.001	0.004
NDF (g/kg DM)	394^bc^	403^de^	398^cd^	408^ef^	401cde	384^a^	414^f^	388^ab^	398^cd^	4.0	0.638	<0.001	<0.001
WSC (g/kg DM)	142^b^	180^de^	196^f^	162^c^	199^f^	217^g^	112^a^	174^d^	186^e^	4.6	<0.001	<0.001	0.007
IVDMD (g/kg DM)	817^bc^	830^cd^	825bcd	809^ab^	828^cd^	835^d^	794^a^	829^cd^	815^bc^	8.2	0.043	<0.001	0.226

IVDMD, *in vitro* dry matter digestibility; SEM, standard error of the mean; OM, organic matter; DM, dry matter; N, nitrogen; NDF, neutral detergent fiber; WSC, water soluble carbohydrates.

a–g Means within the same row with same letters are not significantly different (p>0.05).

**Table 2:** Effects of cutting time and cultivar on *in vitro* fermentation parameters (n = 27)

Items	AberAvon			AberMagic			Premium			SEM	p-value		
			
	AM	Noon	PM	AM	Noon	PM	AM	Noon	PM		Cultivar	Cutting time	Interaction
TGP													
TGP/DM incubated (mL/g DM)	222^ab^	239^cd^	249^e^	232^c^	243^de^	242^de^	217^a^	231^bc^	246^de^	4.2	0.017	<0.001	0.033
TGP/OM incubated (mL/g)	238^ab^	256^d^	265^d^	245^bc^	258^d^	260^d^	232^a^	245^bc^	265^d^	5.2	0.032	<0.001	0.205
TGP/*in vitro* digestible DM incubated (mL/g)	272^a^	287^bc^	302^d^	287^bc^	293^cd^	290bcd	274^a^	278^ab^	302^d^	5.8	0.301	<0.001	0.012
A (mL/g DM)	217^ab^	231^cd^	243^e^	228^cd^	236^de^	237^de^	212^a^	225^bc^	241^e^	2.1	0.022	<0.001	0.029
b (mL/h)	11.5^a^	13.0^c^	12.7^bc^	11.6^a^	12.1^ab^	12.5^bc^	11.3^a^	11.4^a^	11.5^a^	0.14	0.001	0.006	0.152
Lag time (h)	2.89^d^	2.13^bc^	1.75^ab^	2.36^c^	1.66^a^	1.72^ab^	3.40^e^	2.11^bc^	1.63^a^	0.121	0.004	<0.001	0.020
CH_4_													
CH_4_/DM incubated (mL/g DM)	19.2^a^	20.8^bc^	21.0^c^	19.1^a^	19.5^ab^	19.8^ab^	19.3^a^	19.6^ab^	20.4abc	0.63	0.078	0.016	0.516
CH_4_/OM incubated (mL/g)	20.6^ab^	22.3^c^	22.3^c^	20.2^a^	20.7^ab^	21.2abc	20.6^ab^	20.8^ab^	21.7^bc^	0.69	0.059	0.016	0.616
CH_4_/*in vitro* digestible DM incubated (mL/g)	23.5^a^	25.0^ab^	25.4^b^	23.7^a^	23.5^a^	23.7^a^	24.3^ab^	23.6^a^	25.1^ab^	0.82	0.102	0.173	0.281
A (mL/g DM)	18.7^a^	20.3^bc^	20.6^c^	18.7^a^	19.0^a^	19.4abc	18.9^a^	19.1^ab^	20.2^bc^	0.18	0.067	0.003	0.396
b (mL/h)	0.875	0.944	0.941	0.900	0.861	0.893	0.908	0.869	0.860	0.0388	0.393	0.978	0.514
Lag time (h)	4.77^b^	4.16^a^	4.00^a^	5.24^b^	4.05^a^	3.99^a^	5.82^c^	4.77^b^	4.05^a^	0.131	0.002	<0.001	0.081

SEM, standard error of the mean; TGP, total gas production; DM, dry matter; OM, organic matter; A, theoretical maximum of gas production; b, the rate of gas production.

a–e Means within the same row with same letters are not significantly different (p>0.05).

**Table 3:** Relationship between nutrition composition and *in vitro* DM digestibility, and gas production parameters (n = 27)

Items	OM (g/kg DM)	N (g/kg DM)	NDF (g/kg DM)	WSC (g/kg DM)	WSC/N (kg/kg)	WSC/NDF (kg/kg)	NDF/OM (kg/kg)	IVDMD (g/kg DM)
TGP								
TGP/DM incubated (mL/g DM)	-	−0.843***	-	0.823***	0.852***	0.794***	−0.341*	0.489*
TGP/OM incubated (mL/g)	-	−0.853***	−0.400*	0.817***	0.836***	0.791***	−0.354*	0.604***
TGP/*in vitro* digestible DM incubated (mL/g)	-	−0.733***	-	0.640***	0.700***	0.594***	-	-
A (mL/g DM)	-	−0.846***	-	0.808***	0.845***	0.779***	−0.344*	0.463**
b (mL/h)	-	−0.389*	-	0.538**	0.503**	0.548***	-	0.416**
Lag time (h)	-	0.863***	0.458**	−0.889***	−0.903***	−0.869***	0.495**	−0.569**
CH_4_								
CH_4_/DM incubated (mL/g DM)	-	−0.390*	-	0.352*	0.326*	-	-	-
CH_4_/OM incubated (mL/g)	-	−0.408*	-	0.352*	-	-	-	0.331*
CH_4_/*in vitro* digestible DM incubated (mL/g)	-	-	-	-	-	-	-	-
A (mL/g DM)	-	−0.460*	-	0.396*	0.378*	0.353*	-	-
b (mL/h)	-	-	-	-	-	-	-	-
Lag time (h)	-	0.712***	0.477**	−0.826***	−0.806***	−0.812***	0.476**	−0.541*

DM, dry matter; OM, organic matter; N, nitrogen; NDF, neutral detergent fiber; WSC, water soluble carbohydrates; IVDMD, in vitro dry matter digestibility; TGP, total gas production; A, theoretical maximum of gas production; b, the rate of gas production.

- represents no significant relationship.

* p≤0.05,

** p≤0.01,

*** p≤0.001.

**Table 4:** Correlation coefficients between nutrition composition and *in vitro* DM digestibility and gas production parameters with different N levels (N content ≤[n = 15] and >34.0 [n = 12] g/kg DM)

Items	OM (g/kg DM)	N (g/kg DM)	NDF (g/kg DM)	WSC (g/kg DM)	WSC/N (kg/kg)	WSC/NDF (kg/kg)	NDF/OM (kg/kg)	IVDMD (g/kg DM)
N content >34.0 g/kg DM								
TGP								
TGP/DM incubated (mL/g DM)	-	−0.789***	-	0.835***	0.855***	0.825***	-	0.485*
TGP/OM incubated (mL/g)	-	−0.807***	-	0.825***	0.817***	0.800***	-	0.614***
TGP/*in vitro* digestible DM incubated (mL/g)		−0.643***	-	0.636***	0.705***	0.628***	-	-
A (mL/g DM)	-	−0.785***	-	0.812***	0.842***	0.801***	-	0.458**
b (mL/h)	-	−0.528*	-	0.559**	0.542**	0.534***	-	-
Lag time (h)	-	0.799***	-	−0.898***	−0.900***	−0.905***	0.459**	−0.574**
CH_4_								
CH_4_/DM incubated (mL/g DM)	-	-	-	0.423*	-	-	-	-
CH_4_/OM incubated (mL/g)	-	-	-	0.410*	-	-	-	-
CH_4_/*in vitro* digestible DM incubated (mL/g)	-	-	-	-	-	-	-	-
A (mL/g DM)	-	−0.423*	-	0.459*	-	0.419*	-	-
b (mL/h)	-	-	-	-	-	-	-	-
Lag time (h)	-	0.558**	-	−0.795***	−0.735***	−0.800***	0.443*	−0.506*
N content ≤34.0 g/kg DM								
TGP								
TGP/DM incubated (mL/g DM)	-	-	-	−0.690*	−0.737*	−0.625*	-	-
TGP/OM incubated (mL/g)	-	-	-	-	-	-	-	-
TGP/*in vitro* digestible DM incubated (mL/g)			-	−0.726*	−0.863**	−0.838**	-	−0.861**
A (mL/g DM)	-	-	-	−0.685*	−0.703*	−0.607*	-	-
b (mL/h)	-	−0.801**	−0.588*	0.646**	0.650*	0.772**	-	0.758**
Lag time (h)	-	-	-	-	-	-	-	-
CH_4_								
CH_4_/DM incubated (mL/g DM)	-	-	-	-	-	-	-	-
CH_4_/OM incubated (mL/g)	-	-	-	-	-	-	-	-
CH_4_/*in vitro* digestible DM incubated (mL/g)	-	-	-	−0.659*	−0.701*	−0.700*	-	−0.618*
A (mL/g DM)	-	-	-	−0.602*	-	-	-	-
b (mL/h)	-	-	-	-	-	-	-	-
Lag time (h)	-	-	0.477**	-	-	-	-	-

DM, dry matter; OM, organic matter; N, nitrogen; NDF, neutral detergent fiber; WSC, water soluble carbohydrates; IVDMD, in vitro dry matter digestibility; TGP, total gas production; A, theoretical maximum of gas production; b, the rate of gas production.

- represents no significant relationship;

* p≤0.05,

** p≤0.01,

*** p≤0.001.
